# Post-COVID-19 patients in geriatric rehabilitation substantially recover in daily functioning and quality of life

**DOI:** 10.1093/ageing/afae084

**Published:** 2024-05-09

**Authors:** Lisa S van Tol, Miriam L Haaksma, Matteo Cesari, Frances Dockery, Irma H J Everink, Bahaa N Francis, Adam L Gordon, Stefan Grund, Luba Matchekhina, Laura Monica Perez Bazan, Jos M G A Schols, Eva Topinková, Mark A Vassallo, Monique A A Caljouw, Wilco P Achterberg, The EU-COGER consortium, Eva Topinková, Lucie Bautzká, Helena Michaálková, Stefan Grund, Thomas Mross, Lotte Feesche, Rebekka Leonhardt, Clemens Becker, Jan Gerhardus, Brigitte R Metz, Diana Franke-Chowdhury, Rose Galvin, Aoife McCarthy, Frances Dockery, Kara McLoughlin, Bahaa Francis, Matteo Cesari, Annalisa Valentini, Mark Vassallo, Maria Bonnici, Olga Nikolaevna Tkacheva, Ksenia Eruslanova, Luba Matchekhina, Laura Monica Perez Bazan, Esther Roquer Fanlo, Anna Renom Guiteras, Lizzeth Angela Canchucaja, Beatriz Pallardo, Sergio Martínez Zujeros, Margarita Viñuela, Oriol Miralles Resina, Gema Isabel Dominguez, Sarah Caro Bragado, Nadia Stasi, Jennifer Garrillo Cepeda, Marta Arroyo-Huidobro, Ana Gonzalez, Wilco Achterberg, Monique Caljouw, Miriam Haaksma, Lisa van Tol, Saskia Drijver, Paula Vonk, Liesbeth Sikken, Irma Baars, Nathalie Deden, Gerda Nijgh, Sylvia van der Drift, Heike de Wever, Els Calle, Kaoutar Karramass, Josette Hendriks, Lauren Ebbes, Anne Hartman, Hatice Koc, Laura de Vries, Hylco Bouwstra, Laura Langendoen-Wigman, Berber Oldenbeuving, Sabine Noordam-Hemeltjen, Liesbeth Lanting, Lulu Andela, Mathilde Meerkerk, Lianne Willemstein, Krisztina Krasznai, Janneke Wolting, Janette Tazmi, Eveline Keustermans, Janetta de Vries, Sanne van Weers, Lenni Boogaard, Simone Been, Danielle Termeer, Patricia te Pas, Eva Lodewijks, Jeroen van den Berg, Sandra Prent, Marloes Boontje, Joël Harms, Jeffrey Bakker, Carolien de Croon, Christa van Schieveen, Ewout Smit, Patricia van Berlo, Dionne Ruchtie, Jane Manson, Maria Espasandin, Lucy Abbott, Sarah Chadwick, Rebecca Watts, Melani Dani, Jackie McNicholas, Adam Gordon, Vincent Chau

**Affiliations:** Department of Public Health and Primary Care, Leiden University Medical Center, Leiden, The Netherlands; Center for Medicine for Older People, Leiden University Medical Center, Leiden, The Netherlands; University Network for the Care sector South-Holland, Leiden University Medical Center, Leiden, The Netherlands; Department of Public Health and Primary Care, Leiden University Medical Center, Leiden, The Netherlands; Center for Medicine for Older People, Leiden University Medical Center, Leiden, The Netherlands; University Network for the Care sector South-Holland, Leiden University Medical Center, Leiden, The Netherlands; IRCCS Istituti Clinici Maugeri, University of Milan, Milan, Italy; Beaumont Hospital & Royal College of Surgeons in Ireland, Dublin, Ireland; Department of Health Services Research, Maastricht University, Maastricht, The Netherlands; Fliman Geriatric Rehabilitation Hospital, Zalman Shneur Street, Haifa, 31021, Israel; Geriatric Division, Holy Family Hospital, Bar Ilan University, Safad, Israel; Academic Unit of Injury, Recovery and Inflammation Sciences (IRIS), School of Medicine, University of Nottingham, Medical School, Nottingham, NG7 2UH, UK; Center for Geriatric Medicine, Agaplesion Bethanien Hospital Heidelberg, Geriatric Center at the Heidelberg University, Heidelberg, Germany; Russian Gerontology Research and Clinical Centre, Pirogov Russian National Research Medical University, Moscow, Russia; RE-FiT Barcelona Research Group, Parc Sanitari Pere Virgili Hospital and Vall d’Hebron Institut de Recerca (VHIR), Barcelona, Spain; Department of Health Services Research, Maastricht University, Maastricht, The Netherlands; Department of Geriatrics, First Faculty of Medicine, Charles University and General Faculty Hospital, Prague, Czech Republic; Faculty of Health and Social Sciences, University of South Bohemia, Ceske Budejovice, Czech Republic; Karin Grech Hospital, Pieta, Malta; Department of Public Health and Primary Care, Leiden University Medical Center, Leiden, The Netherlands; Center for Medicine for Older People, Leiden University Medical Center, Leiden, The Netherlands; University Network for the Care sector South-Holland, Leiden University Medical Center, Leiden, The Netherlands; Department of Public Health and Primary Care, Leiden University Medical Center, Leiden, The Netherlands; Center for Medicine for Older People, Leiden University Medical Center, Leiden, The Netherlands; University Network for the Care sector South-Holland, Leiden University Medical Center, Leiden, The Netherlands

**Keywords:** geriatric rehabilitation, COVID-19, recovery, older people

## Abstract

**Background:**

After an acute infection, older persons may benefit from geriatric rehabilitation (GR).

**Objectives:**

This study describes the recovery trajectories of post-COVID-19 patients undergoing GR and explores whether frailty is associated with recovery.

**Design:**

Multicentre prospective cohort study.

**Setting:**

59 GR facilities in 10 European countries.

**Participants:**

Post-COVID-19 patients admitted to GR between October 2020 and October 2021.

**Methods:**

Patients’ characteristics, daily functioning (Barthel index; BI), quality of life (QoL; EQ-5D-5L) and frailty (Clinical Frailty Scale; CFS) were collected at admission, discharge, 6 weeks and 6 months after discharge. We used linear mixed models to examine the trajectories of daily functioning and QoL.

**Results:**

723 participants were included with a mean age of 75 (SD: 9.91) years. Most participants were pre-frail to frail (median [interquartile range] CFS 6.0 [5.0–7.0]) at admission. After admission, the BI first steeply increased from 11.31 with 2.51 (SE 0.15, *P* < 0.001) points per month and stabilised around 17.0 (quadratic slope: −0.26, SE 0.02, *P* < 0.001). Similarly, EQ-5D-5L first steeply increased from 0.569 with 0.126 points per month (SE 0.008, *P* < 0.001) and stabilised around 0.8 (quadratic slope: −0.014, SE 0.001, *P* < 0.001). Functional recovery rates were independent of frailty level at admission. QoL was lower at admission for frailer participants, but increased faster, stabilising at almost equal QoL values for frail, pre-frail and fit patients.

**Conclusions:**

Post-COVID-19 patients admitted to GR showed substantial recovery in daily functioning and QoL. Frailty at GR admission was not associated with recovery and should not be a reason to exclude patients from GR.

## Key Points

- Post-COVID-19 patients from geriatric rehabilitation (GR) centres across 10 European countries showed substantial recovery.
- Recovery in daily functioning and quality of life was independent of frailty level at admission to GR following COVID-19.
- Frailty should not be a reason to exclude patients from GR, as even frail people may considerably benefit from post-acute care.

## Introduction

The COVID-19 pandemic was associated with millions of severe acute respiratory syndrome coronavirus 2 (SARS-CoV-2) infections and deaths worldwide, but the highest infection rates and most severe infections were among older people [1–5]. Older people with SARS-CoV-2 infection were more often admitted to hospital and to Intensive Care Unit (ICU), with periods of immobility as a consequence [6].

Under normal circumstances, older people experiencing acute deterioration in their health and functional status would be offered geriatric rehabilitation (GR) [7–9]. GR is aimed at people with complex health problems, including pre-existing multimorbidity, cognitive impairment, frailty or other geriatric syndromes [10]. GR can be provided in diverse care settings [11]. During the COVID-19 pandemic, the availability of GR care was diminished due to illness among staff, secondment to acute care wards, repurposing of GR facilities as isolation beds for SARS-CoV-2-positive patients and reduced capacity due to pandemic-related spacing requirements [12]. This reduction in rehabilitation supply at a time when demand increased due to many older people experiencing acute health deteriorations due to COVID-19 has been called the ‘COVID-19 rehabilitation paradox’ [12, 13].

Future pandemic planning should include more effective provision of rehabilitation. Therefore, we need to know whether GR is successful in this context, what type of rehabilitation care to deliver and what population sub-groups are likely to benefit [10, 14]. Evidence on recovery trajectories for people in GR post-COVID is still limited, but suggests that people participating in GR post-COVID experienced at least partial recovery [15–18]. Outside the context of COVID-19, frailty and functional decline are both frequently used criteria in triage of acutely hospitalised patients for referral to GR [19]. Moreover, frailty in older people has been associated with lower functional status [20, 21] and quality of life (QoL) [22]. Against this background, in this study we aim to describe the recovery trajectories in daily functioning and QoL of geriatric patients after COVID-19 in a multicentre, multinational European cohort during GR and up to 6 months after discharge; and explore whether the patient’s frailty level at GR admission is associated with recovery in daily functioning and QoL.

## Methods

### Design

The European Cooperation in Geriatric Rehabilitation study after COVID-19 (EU-COGER) was an international multicentre prospective observational cohort study. This study was designed by the Special Interest Group for Geriatric Rehabilitation of the European Geriatric Medical Society (EuGMS) and registered in ClinicalTrials.gov (identifier: NCT05749731)*.*

### Participants and setting

The terminology and definitions used for GR differ between countries. In this study, we defined GR facilities, in line with the consensus definition for GR developed by the EuGMS [11], as facilities that provide multidisciplinary rehabilitation care to frail and/or multimorbid patients. Participants were recruited from the Czech Republic, Germany, Ireland, Israel, Italy, Malta, Russia, Spain, the Netherlands, and the UK between October 2020 and October 2021 [7]. Both inpatient GR facilities and GR facilities that provided care at home were included in the EU-COGER consortium (Appendix I).

To be included, patients had to be receiving rehabilitation in one of the participating facilities as part of recovery from a SARS-CoV-2 infection, confirmed with either polymerase chain reaction for viral RNA or serology for antibodies against SARS-CoV-2. Potential participants with severe cognitive impairment that led to insufficient decisional capacities to participate in the study were excluded [7].

### Ethics

The Leiden University Medical Center COVID-19 science ethical committee deemed this study exempt from the Medical Research Involving Human Subjects Act (Wet medisch-wetenschappelijk onderzoek met mensen, WMO), since the study only used routinely collected data, and approved the study based on an opt-out procedure for the Netherlands (protocol number CoCo 2020-040). In all other countries, the local ethical regulations were followed and approval from local ethics committee was granted as per local regulations.

### Data collection

Routine medical care data from health records were collected at admission to GR, and at discharge, including data from 2 weeks premorbid (pre-COVID) status from admission documentation [7]. In addition, participating facilities were asked to collect data through telephone follow-ups at 6 weeks and 6 months after discharge. Local study coordinators entered participant data into an online CASTOR [23] database using standard operating procedures [24]. A complete overview of the procedures and all measures collected is described in the published protocol paper [7].

Outcome measures chosen were based on instruments readily available in multiple languages and cross-culturally validated. The primary outcome measure was daily functioning, assessed with the Barthel Index (BI) for activities of daily living (ADL) at all time points [25]. When certain countries or facilities used comparable measures, i.e. the Utrecht Scale for the Evaluation of Rehabilitation or the Functional Independence Measure, these were converted to the BI using standardised approaches [26, 27]. The BI is a 10-item instrument that produces a total score that ranges from 0 to 20, with higher scores indicating higher independence in ADL.

The secondary outcome measure was health-related QoL assessed with the EQ-5D-5L, available in >150 languages [28]*.* EQ-5D-5L was assessed at all timepoints except premorbid, and is a 5-item instrument that produces a 5-digit status for mobility, self-care, daily activities, pain and anxiety/depression. Using an available country tariff, this status can be calculated into a societal value of maximum 1 for optimal QoL [29–34]. Malta, Czech Republic and Russia had no country tariff available, and the geographically closest available country tariff (Spain, Poland and Poland, respectively) was used [32, 35]. There was no QoL data available for Israeli participants, as data necessary for EQ-5D-5L were not collected as part of routine practice.

Frailty, the independent variable of interest, was measured using the Clinical Frailty Scale (CFS). This ranks frailty on a scale from level 1 to 9, with level 1 ‘very fit’ to 9 ‘terminally ill’ [36]. Premorbid frailty level and frailty level at GR admission were collected. Other variables collected include demographic characteristics, clinical characteristics and received treatment components (Table 1).

**Table 1:** Demographic characteristics, clinical characteristics and received treatment components and outcomes of post-COVID-19 patients in geriatric rehabilitation

Characteristic	*n* (%) available	Value
Age, mean (SD)	719 (99.4)	75.49 (9.91)
Sex, male, *n* (%)	723 (100)	379 (52.4)
Country, *n* (%)	723 (100)	
Czech Republic		53 (7.3)
Germany		50 (6.9)
Ireland		50 (6.9)
Israel		32 (4.4)
Italy		30 (4.1)
Malta		17 (2.4)
Russia		50 (6.9)
Spain		96 (13.3)
The Netherlands		293 (40.6)
UK		52 (7.2)
Barthel Index at GR admission, mean (SD)	714 (98.8)	10.94 (5.40)
EQ-5D-5L at GR admission, mean (SD)	471 (65.1)	0.52 (0.32)
Clinical Frailty Scale (CFS) premorbid, median (IQR)	490 (67.8)	3.0 (2.0–4.0)
Fit (CFS 1–3)		283 (39.1)
Pre-frail (CFS 4–5)		149 (20.6)
Frail (CFS 6–9)		58 (8.0)
Clinical Frailty Scale (CFS) at GR admission, median (IQR)	493 (68.2)	6.0 (5.0–7.0)
Fit (CFS 1–3)		51 (7.1)
Pre-frail (CFS 4–6)		129 (17.8)
Frail (CFS 7–9)		313 (43.3)
Functional Comorbidity Index, median (IQR)	634 (87.7)	3.0 (2.0–4.0)
Hospital stay preadmission, *n* (%)	720 (99.6)	653 (90.3)
Hospital length of stay, days, median (IQR)	645 (89.2)	23.0 (13.0–46.5)
ICU stay preadmission, yes, *n* (%)	711 (98.3)	240 (33.2)
ICU length of stay, days, median (IQR)	232 (32.1)	23.0 (11.0–43.0)
Living situation premorbid, *n* (%)	720 (99.6)	
Own home		675 (93.4)
Nursing home/assisted living		42 (5.8)
Other		3 (0.4)
Treatment components of GR, *n* (%)	670 (92.7)	
Oxygen therapy		289 (40.0)
Physiotherapy (total)		595 (82.3)
Physiotherapy for sarcopenia		496 (74.0)
Physiotherapy for lung function		408 (60.9)
Occupational therapy (total)		467 (64.6)
Occupational therapy for iADL		421 (62.8)
Occupational therapy for house adaptations		273 (40.7)
Speech/language therapy (total)		126 (17.4)
Speech/language therapy for dysphagia		93 (13.9)
Speech/language therapy for voice/speech		61 (9.1)
Protein- or calorie-enriched diet		437 (60.4)
Psychosocial support		170 (23.5)
Cognitive training		82 (11.3)
Length of stay in GR, days; median (IQR)	701 (97.0)	26.0 (15.0–41.0)
Discharge destination, % (*n*)	703 (97.2)	
Own home		544 (75.2)
Nursing home/assisted living		103 (14.3)
Hospital		30 (4.1)
Other		15 (2.1)
Deceased during GR		11 (1.5)
Post-traumatic stress disorder at 6 weeks and/or 6 months after GR discharge, % (*n*)	541 (74.8)	59 (8.16)

### Statistical analysis

Descriptive statistics were used to give an overview of participants’ demographic and clinical characteristics, and treatment components. Continuous variables were reported with mean and SD or median and interquartile range (IQR), depending on whether data were normally distributed. Categorical variables were presented as number (*n*) and percentage (%).

The recovery trajectories in daily functioning and QoL during and after GR were examined by linear mixed models, with time in months since GR admission. For each outcome measure, three models were built. Unconditional models were used to illustrate the change in daily functioning and QoL of the study population over time, independent of covariates. To identify the best fitting unconditional models, the following steps were taken: first, we tested whether the fixed slopes were linear or quadratic; second, we tested whether adding random intercept parameters for variance between persons and between countries improved the model fit; third, we tested whether adding random linear and quadratic slope parameters for variance between persons and variance between countries improved the model fit. In every step, we fitted models using the default optimizer in the lmer R function ‘nloptwrap’, and optimizer ‘Neldermead’ that has been specially developed to find solutions of boundary fits [37]. The model with the highest loglikelihood value (for nested models) or the lowest Akaike Information Criterion value (for non-nested models) was chosen [38]. Models were built with unstructured variance–covariance matrices. For daily functioning, a premorbid value was available, but not for QoL. Therefore, we were able to add a linear spline from premorbid to GR admission in the models for daily functioning.

Subsequently, the effect of frailty at GR admission on recovery in daily functioning and QoL was examined in univariable models and in multivariable models adjusted for age, sex, premorbid daily functioning, comorbidities (Functional Comorbidity Index) [39], hospital length of stay (days) and ICU stay (yes/no). All independent variables were mean-centred to present the recovery trajectory in daily functioning and QoL for a sample mean participant. In the same way, the effect of premorbid frailty on trajectories of daily functioning and QoL was examined in a sensitivity analysis. In addition, we tested whether participants with missing values in the independent variables, who had to be excluded from complete case analysis, had similar recovery trajectories as the included participants.

Outcomes were presented as parameter estimates (SE) for the fixed and random effects of the mixed models. All models were built using R version 4.2.2 and R function lmer for linear mixed models from R package lme4. The effect of frailty is illustrated in graphs for three stages of frailty defined as fit (CFS 1–3), pre-frail (CFS 4–5) and frail (CFS 6–9) [40, 41].

## Results

### Participants

**Figure 1:**
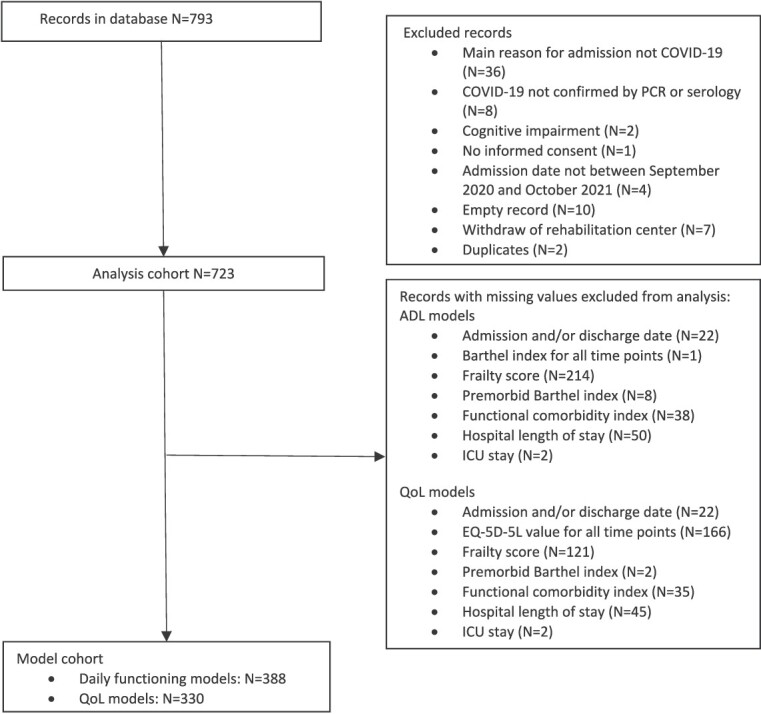
Flowchart of study participants.

Participants were recruited from 59 rehabilitation facilities in 10 European countries. Records for 793 participants were created in the database. After the exclusion of participants from rehabilitation centres that withdrew from study participation (*n* = 7), duplicates (*n* = 2), empty records (*n* = 10) and participants who did not meet the inclusion criteria (*n* = 51), the cohort consisted of 723 participants (Figure 1).

Participants’ mean age was 75 years (SD 9.9), and most of them had been admitted to the hospital prior to GR (*n* = 653; 90.3%). While premorbid most participants were fit or pre-frail (*n* = 490, CFS 3.0, IQR 2.0–4.0), at GR admission most participants were frail or pre-frail (*n* = 493, median CFS 6.0, IQR 5.0–7.0). More than half of the participants received physiotherapy (88.9%), occupational therapy (69.7%), and a protein- or calorie-enriched diet (65.3%) during GR. The median length of stay in GR was 26.0 days (IQR 15.0–41.0) (Table 1). The available numbers of daily functioning and QoL scores for each timepoint are presented in Appendix II.

Data of respectively 388 and 330 participants were complete for all covariates and had outcome data for at least one of the timepoints and could be included in the linear mixed models (Figure 1). There were no clinically relevant differences between the recovery trajectories for participants included and excluded from the models for daily functioning.

### Daily functioning over time

**Figure 2:**
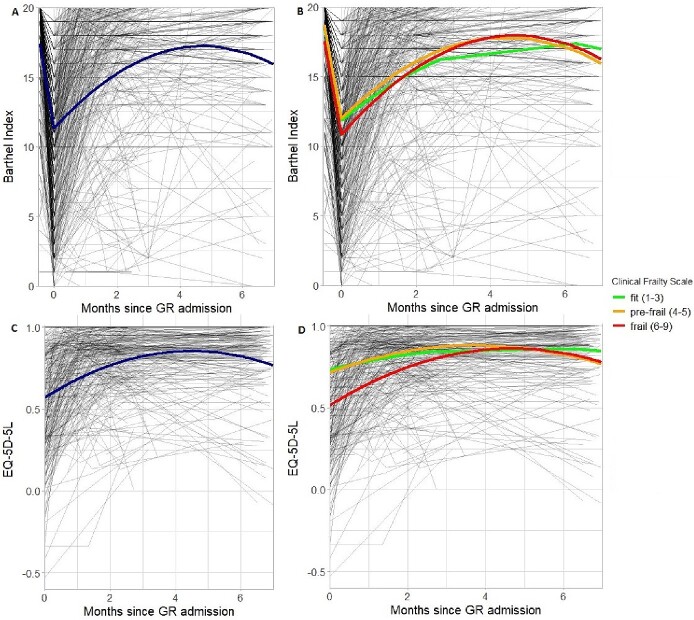
**A**, Unconditional trajectory of daily functioning (*n* = 388). **B**, Trajectory of daily functioning for fit (*n* = 34), pre-frail (*n* = 102) and frail (*n* = 252) participants at GR admission (*n* = 388). **C**, Unconditional trajectory of quality of life (*n* = 330). **D**, Trajectory of quality of life for fit (*n* = 33), pre-frail (*n* = 95) and frail (*n* = 202) participants at GR admission (*n* = 330).

The best fitting unadjusted model for the recovery trajectory of daily functioning showed that BI decreased during acute COVID infection from 17.41 before GR admission to 11.31 BI (SE 0.81, *P* < 0.001; Table 2) at GR admission. After GR admission, the largest increase in BI was seen within the first 3 months: BI first steeply increased with 2.51 (SE 0.18, *P* < 0.001) points BI per month and stabilised (quadratic slope: −0.26 BI per month squared, SE 0.02, *P* < 0.001) around 17.0 (Figure 2A). This best fitting model contained random intercepts and slopes for participants and countries.

**Table 2:** Linear mixed models for change in daily functioning over time (unconditional model) and effect of frailty (univariable and multivariable models) (*n* = 388)

	Unadjusted model		Univariable model		Multivariable model^*^	
	Estimate (SE)	*P*-value	Estimate (SE)	*P*-value	Estimate (SE)	*P*-value
**Fixed effects**						
*At admission (intercept)*						
Daily functioning (Barthel Index; range 0–20)	11.31 (0.81)	<0.001	11.51 (0.46)	<0.001	11.64 (0.31)	<0.001
Frailty (Clinical Frailty Scale; range 1–9)	N/A	N/A	−1.50 (0.13)	<0.001	−0.90 (0.11)	<0.001
*Change before admission (slope)*						
Change per week	−3.05 (0.11)	<0.001	−3.08 (0.11)	<0.001	−3.10 (1.06)	<0.001
*Change after admission (slope)*						
Per month: linear component	2.51 (0.15)	<0.001	2.58 (0.15)	<0.001	2.73 (0.14)	<0.001
Per frailty score: linear component	N/A	N/A	−0.13 (0.09)	0.170	−0.17 (0.09)	0.075
Per month: quadratic component	−0.26 (0.02)	<0.001	−0.28 (0.02)	<0.001	−0.30 (0.02)	<0.001
Per frailty score: quadratic component	N/A	N/A	0.03 (0.01)	0.019	0.04 (0.01)	0.007
	Variance (SD)		Variance (SD)		Variance (SD)	
**Random effects**						
*At admission (intercept)*						
Between persons variance	8.13 (2.85)		5.40 (2.32)		1.50 (1.23)	
Between countries variance	6.00 (2.45)		1.72 (1.31)		0.70 (0.84)	
*After admission (slope of change)*						
Between persons variance	0.03 (0.19)		0.04 (0.20)		0.05 (0.22)	
Between countries variance	0.07 (0.26)		0.05 (0.22)		0.01 (0.10)	
*Residual*	10.64 (3.26)		10.52 (3.24)		9.98 (3.16)	

N/A, not available.

^*^Adjusted for age, sex, premorbid BI, Functional Comorbidity Index, hospital length of stay and ICU stay.

The multivariate model showed that BI for daily functioning at GR admission was significantly lower for participants who were frailer at GR admission, estimated as 0.90 (SE 0.11, *P* < 0.001) points lower BI for each point that CFS is higher (Table 2). Frailty at GR admission had little effect on the rate of recovery in daily functioning (linear slope: −0.17 points BI per point CFS per month, SE 0.09, *P* = 0.075; quadratic slope 0.04 points BI per point CFS per month squared, SE 0.01, *P* = 0.007). Figure 2B shows that the recovery trajectories of daily functioning for participants of different frailty stages were almost parallel to each other.

Sensitivity analysis showed that premorbid frailty had a stronger association with the rate of recovery in daily functioning compared to frailty at GR admission. Participants who were frail premorbid (CFS 6–9, *n* = 49) recovered more slowly, leading to only partial recovery in daily functioning (Appendix III: Table 5, Figure 3A).

### Quality of life over time

The best fitting unadjusted model for the recovery trajectory of QoL showed that the largest increase was seen within the first 2 months: The EQ-5D-5L value also first steeply increased from 0.569 (SE 0.047, *P* < 0.001; Table 3) at GR admission with 0.126 (SE 0.008, *P* < 0.001) per month, after which it stabilised (quadratic slope: −0.014 points EQ-5D-5L per month squared, SE 0.001, *P* < 0.001) around 0.8 (Figure 2C). This best fitting model contained random intercepts for participants and countries and a random slope for participants.

**Table 3:** Linear mixed models for change in quality of life over time (unconditional model) and effect of frailty (univariable and multivariable models (*n* = 330))

	Unadjusted model		Univariable model		Multivariable model^*^	
	Estimate (SE)	*P*-value	Estimate (SE)	*P*-value	Estimate (SE)	*P*-value
**Fixed effects**						
*At admission (intercept)*						
Quality of life (EQ-5D-5L; range 0–1)	0.569 (0.047)	<0.001	0.566 (0.037)	<0.001	0.587 (0.031)	<0.001
Frailty (Clinical Frailty Score; range 1–9)	N/A	N/A	−0.098 (0.01)	<0.001	−0.075 (0.010)	<0.001
*Change after admission (slope)*						
Per month: linear component	0.126 (0.008)	<0.001	0.125 (0.008)	<0.001	0.124 (0.008)	<0.001
Per frailty score: linear component	N/A	N/A	0.027 (0.006)	<0.001	0.023 (0.007)	<0.001
Per month: quadratic component	−0.014 (0.001)	<0.001	−0.014 (0.001)	<0.001	−0.014 (0.001)	<0.001
Per frailty score: quadratic component	N/A	N/A	−0.003 (0.001)	0.006	−0.002 (0.001)	0.033
	Variance (SD)		Variance (SD)		Variance (SD)	
**Random effects**						
*At admission (intercept)*						
Between persons variance	0.035 (0.187)		0.026 (0.160)		0.022 (0.148)	
Between countries variance	0.018 (0.132)		0.011 (0.103)		0.007 (0.082)	
*After admission (slope of change)*						
Between persons variance	0.001 (0.032)		0.001 (0.029)		0.001 (0.025)	
*Residual*	0.030 (0.172)		0.029 (0.171)		0.029 (0.170)	

N/A, not available.

^*^Adjusted for age, sex, premorbid BI, Functional Comorbidity Index, hospital length of stay and ICU stay.

The multivariate model found that EQ-5D-5L values for QoL at GR admission were much lower for participants who were frailer at GR admission, estimated as 0.07 (SE 0.01, *P* < 0.001) points lower EQ-5D-5L for each point that CFS is higher (Table 3). Frailty at GR admission was also associated with the rate of recovery in daily functioning. EQ-5D-5L values increased steeper for frailer participants (linear slope 0.02 higher EQ-5D-5L value per point CFS per month, SE 0.01, *P* < 0.001; quadratic slope <−0.00 lower EQ-5D-5L value per point CFS per month squared, SE 0.00, *P* = 0.033). Figure 2D shows that within some months this led to almost equal EQ-5D-5L values for frail, pre-frail and fit participants.

Sensitivity analysis showed that the association between premorbid frailty and the rate of recovery in QoL was similar to the association for frailty at GR admission: the rate of recovery in QoL was higher for frailer participants (Appendix III: Table 6, Figure 3B).

## Discussion

This study showed that European patients admitted to GR following COVID-19 recovered in daily functioning almost up to their premorbid status. Their QoL also substantially increased. The largest increases in QoL and daily functioning were observed within the first 2 or 3 months after GR admission. A large proportion of geriatric post-COVID-19 patients were frail at GR admission. These frail patients recovered in daily functioning approximately as fast as more fit patients. Although QoL was lower at admission for patients who were frail (either at GR admission or prior to the infection), their recovery went faster compared to fitter patients, leading to equal levels of QoL after a couple of months.

This study was performed during a period when healthcare systems were severely strained, and this likely reduced the quality of rehabilitation care. Patients were sometimes discharged early from the hospital [42]. Consequently, possibly patients were frailer than usual at GR admission. Therefore, the observed recovery may be an underestimation of the potential recovery of post-COVID-19 patients. Moreover, post-COVID-19 GR patients in our cohort (mean age 75, SD 9.9) tend to be a little younger than pre-pandemic GR patients (mostly patients recovering from stroke, complex conditions, hip fracture or repeated falls), who have a mean age of 80 (SD 4.3) [43].

Literature about older COVID-19 patients who did not receive rehabilitation care after hospitalisation shows that the majority of them did not fully recover. In a French and in a Spanish cohort, one-third had a lower functional status at 3 months after hospitalisation than they had at hospital admission [44, 45]. Moreover, the majority experienced cognitive decline, depressive symptoms, required readmission or died [44]; or experienced fatigue, frailty or died [45]. Two-thirds of the older post-COVID-19 patients in a Norwegian cohort reported a decline in any of the EQ-5D-5L dimensions from their premorbid situation to 6 months after hospital discharge [46]. Frailty, either measured premorbid or at hospital admission, has been shown to be associated with mortality in hospitalised older people with COVID-19 [41, 47, 48].

The present study found that for patients who were admitted to GR, frailty at admission was not distinctive for recovery. Even patients who were frail premorbid partially recovered, though less completely so (Appendix III). These findings support inclusivity when selecting patients for GR. Guidelines are ambiguous about the use of frailty as a selection criterion for GR after COVID-19. For example, according to guidance by the EuGMS, a geriatric needs assessment, which includes frailty, should be used in the referral decision [10]. Guidelines developed by the World Health Organisation do not mention frailty as a criterion for GR referral [49]. Instead, these guidelines describe that rehabilitation programmes should be individualised based on functional limitations [49].

This study has a number of strengths. First, to our knowledge, this is the only study on COVID-19 rehabilitation with a follow-up time of >6 months. Second, patients were recruited from 59 rehabilitation facilities in 10 European countries. However, in the Czech Republic, Italy, Israel and Malta, only one care facility participated, which may reduce the generalisability of our results in these countries. Third, this study specifically focussed on GR. Little research has been done on COVID-19 in this field.

A limitation of this study is the lack of more detailed outcome measures, such as instrumental ADL (iADL), because only regular care data were collected. Second, few participants were fit at GR admission (*n* = 51), and few participants were frail prior to the SARS-COV-2 infection (*n* = 58). Therefore, our results are not very precise for these patient groups. However, this is unlikely to pose large threat to the generalisability of our findings to the GR population, as GR patients are often selected based on their potential to benefit from GR, leading to relatively small numbers of premorbid frail or at admission very fit patients. Third, a large number of participants had to be excluded from the linear mixed models due to missing values. However, it is unlikely that this biassed our results. Mixed models handle missing outcome data well under the assumption it is missing at random, and the recovery trajectories of the excluded and included participants were similar. Fourth, due to the wide practice variation, it is unclear whether our results apply to all GR care settings and what optimal GR care constitutes.

In conclusion, this study found that patients admitted to GR following COVID-19 substantially recover in terms of daily functioning and QoL. Even patients who were frail at GR admission substantially recovered, which suggests that post-COVID-19 patients of all stages of frailty have the potential to benefit from GR care and that frailty after acute illness should not be used as a criterion to decline patients access to rehabilitation. However, more research is needed to quantify the association between premorbid frailty and rehabilitation potential. To make statements about what optimal GR care for post-COVID-19 patients constitutes, differences between countries in GR care organisation, patient selection and recovery trajectories should be explored. Barring a deterioration in the current global situation regarding COVID-19, opportunities to conduct similar large-scale research in this context are unlikely to arise. The work presented here may be extrapolated to other contexts and acute conditions with similar clinical trajectories to bring our understanding forward of where GR may add value.

## Supplementary Material

aa-23-1978-File003_afae084
