# Supplementary material for: Post-COVID-19 patients in geriatric rehabilitation substantially recover in daily functioning and quality of life
Source: Age Ageing. 2024 May 9;53(5):afae084. doi: 10.1093/ageing/afae084 (PMC11082471; doi:10.1093/ageing/afae084)
Supplement: aa-23-1978-File003_afae084 [file aa-23-1978-file003_afae084.docx]

| **Appendix I. EU-COGER consortium list** | | | | |  |
| --- | --- | --- | --- | --- | --- |
| **Name of health centre** | **Country** | **Study coordinator #1** | **Study coordinator #2** | **Study coordinator #3** | **Study coordinator #4** |
| Vseobecna fakultní nemocnice | Czech Republic | Eva Topinková | Lucie Bautzká | Helena Michaálková |  |
| Agaplesion Bethanien Hospital | Germany | Stefan Grund | Thomas Mross | Lotte Feesche |  |
| Robert-Bosch-Krankenhaus | Germany | Rebekka Leonhardt | Clemens Becker |  |  |
| Geriatrisches Zentrum Karlsruhe | Germany | Jan Gerhardus | Brigitte R. Metz |  |  |
| Geriatrische Rehabilitationsklinik Diakonissenkrankenhaus Mannheim | Germany | Diana Franke-Chowdhury |  |  |  |
| University of Limerick Hospital Group (ULHG) | Ireland | Rose Galvin | Aoife McCarthy |  |  |
| Beaumont Hospital | Ireland | Frances Dockery | Kara McLoughlin |  |  |
| Fliman geriatric rehabilitation center | Israel | Bahaa Francis |  |  |  |
| IRCCS Istituti Clinici Maugeri | Italy | Matteo Cesari | Annalisa Valentini |  |  |
| Karin Grech Hospital | Malta | Mark Vassallo | Maria Bonnici |  |  |
| Russian Clinical and Research Center of Gerontology | Russia | Olga Nikolaevna Tkacheva | Ksenia Eruslanova |  |  |
| Moscow Rehabilitation center | Russia | Luba Matchekhina |  |  |  |
| Parc Sanitari Pere Virgili | Spain | Laura Monica Perez Bazan |  |  |  |
| Hospital Universitari Sant Joan de Reus | Spain | Esther Roquer Fanlo |  |  |  |
| Hospital Universitari Parc de Salut Mar | Spain | Anna Renom Guiteras | Lizzeth Angela Canchucaja |  |  |
| Hospital Central de la Cruz Roja San José y Santa Adela | Spain | Beatriz Pallardo | Sergio Martínez Zujeros |  |  |
| Hospital San Joan de Deu Mallorca | Spain | Margarita Viñuela | Oriol Miralles Resina |  |  |
| Hospital Guadarrama | Spain | Gema Isabel Dominguez | Sarah Caro Bragado |  |  |
| Hospital de Barcelona | Spain | Nadia Stasi | Jennifer Garrillo Cepeda |  |  |
| Consorci Sanitari Alt’Pènedes i Garraf | Spain | Marta Arroyo-Huidobro | Ana Gonzalez |  |  |
| Leiden University Medical Center | the Netherlands | Wilco Achterberg | Monique Caljouw | Miriam Haaksma | Lisa van Tol |
| Omring | the Netherlands | Saskia Drijver |  |  |  |
| Zorgcirkel | the Netherlands | Paula Vonk |  |  |  |
| BrabantZorg | the Netherlands | Liesbeth Sikken | Irma Baars |  |  |
| Ijsselheem | the Netherlands | Nathalie Deden |  |  |  |
| Topaz Revitel | the Netherlands | Gerda Nijgh | Sylvia van der Drift |  |  |
| Tante Louise | the Netherlands | Heike de Wever | Els Calle |  |  |
| MUMC+\|Herstelzorg – Vitala+ | the Netherlands | Kaoutar Karramass | Josette Hendriks |  |  |
| Axion continu | the Netherlands | Lauren Ebbes |  |  |  |
| TriviumMeulenbeltZorg Almelo | the Netherlands | Anne Hartman | Hatice Koc |  |  |
| TriviumMeulenbeltZorg Hengelo | the Netherlands | Laura de Vries |  |  |  |
| Patyna | the Netherlands | Hylco Bouwstra |  |  |  |
| Careyn | the Netherlands | Laura Langendoen-Wigman |  |  |  |
| Sensire | the Netherlands | Berber Oldenbeuving | Sabine Noordam-Hemeltjen |  |  |
| Azora | the Netherlands | Liesbeth Lanting | Lulu Andela |  |  |
| Argos Zorggroep | the Netherlands | Mathilde Meerkerk |  |  |  |
| Meriant (Alliade) | the Netherlands | Lianne Willemstein | Krisztina Krasznai |  |  |
| Liemerije | the Netherlands | Janneke Wolting |  |  |  |
| Laurens Intermezzo Zuid | the Netherlands | Janette Tazmi |  |  |  |
| de Wever | the Netherlands | Eveline Keustermans |  |  |  |
| Icare – De Boshof | the Netherlands | Janetta de Vries | Sanne van Weers |  |  |
| SVRZ ‘t Gasthuis | the Netherlands | Lenni Boogaard |  |  |  |
| De Betuwe, Zorgcentrum Beatrix | the Netherlands | Simone Been |  |  |  |
| Archipel Zorggroep | the Netherlands | Danielle Termeer |  |  |  |
| Florence | the Netherlands | Patricia te Pas | Eva Lodewijks |  |  |
| Pieter van Foreest, locatie Bieslandhof | the Netherlands | Jeroen van den Berg |  |  |  |
| Reactiveringscentrum Klimop | the Netherlands | Sandra Prent | Marloes Boontje |  |  |
| Zorgspectrum Nieuwegein | the Netherlands | Joël Harms | Jeffrey Bakker |  |  |
| Zorggroep Maas en Waal | the Netherlands | Carolien de Croon |  |  |  |
| Attent | the Netherlands | Christa van Schieveen |  |  |  |
| Vivium Flevoburen (Zorggroep Almere) | the Netherlands | Ewout Smit |  |  |  |
| Kennemerhart Schoterhof | the Netherlands | Patricia van Berlo |  |  |  |
| Van Neynsel | the Netherlands | Dionne Ruchtie |  |  |  |
| Sheffield teaching Hospitals | UK | Jane Manson |  |  |  |
| Frimley Health NHS Foundation Trust | UK | Maria Espasandin | Lucy Abbott |  |  |
| Harrogate District Hospital | UK | Sarah Chadwick | Rebecca Watts |  |  |
| Imperial College Healthcare NHS Trust | UK | Melani Dani | Jackie McNicholas |  |  |
| University Hospitals of Derby and Burton | UK | Adam Gordon |  |  |  |
| Calderdale & Huddersfield | UK | Vincent Chau |  |  |  |
| Derbyshire Community Health Services | UK | Andy Cole |  |  |  |

**Appendix II. Availability of ADL functioning and quality of life data per timepoint**

| **Table 4. availability of ADL functioning and quality of life data** | |
| --- | --- |
| **Outcome measures** | **n (%) available** |
| ADL functioning (Barthel index) |  |
| premorbid | 641 (88.7) |
| GR admission | 714 (98.8) |
| GR discharge | 655 (90.6) |
| 6 weeks after discharge | 515 (71.2) |
| 6 months after discharge | 509 (70.4) |
|  |  |
| Quality of life (EQ-5D-5L) |  |
| GR admission | 471 (65.1) |
| GR discharge | 413 (57.1) |
| 6 weeks after discharge | 423 (58.5) |
| 6 months after discharge | 425 (58.8) |

**Appendix III: ADL functioning and quality of life over time and the effect of premorbid frailty on these trajectories**

| Table 5. Linear mixed models for change in ADL functioning over time (unconditional model) and effect of premorbid frailty (univariable and multivariable models) (n=389) | | | | | | |
| --- | --- | --- | --- | --- | --- | --- |
|  | Unadjusted model | | Univariable model | | Multivariable model* | |
|  | Estimate (SE) | p-value | Estimate (SE) | p-value | Estimate (SE) | p-value |
| **Fixed effects** |  |  |  |  |  |  |
| At admission (intercept) |  |  |  |  |  |  |
| ADL functioning (Barthel Index; range 0-20) | 11.35 (0.82) | <0.001 | 11.73 (0.51) | <0.001 | 11.65 (0.52) | <0.001 |
| frailty (Clinical Frailty Scale; range 1-9) | N/A | N/A | -1.45 (0.12) | <0.001 | -0.29 (0.14) | 0.036 |
|  |  |  |  |  |  |  |
| Change before admission (slope) |  |  |  |  |  |  |
| Change per week | - 3.06 (0.11) | <0.001 | - 3.06 (0.11) | <0.001 | - 3.10 (1.06) | <0.001 |
|  |  |  |  |  |  |  |
| Change after admission (slope) |  |  |  |  |  |  |
| Per month: linear component | 2.45 (0.15) | <0.001 | 2.47 (0.15) | <0.001 | 2.65 (0.14) | <0.001 |
| Per frailty score: linear component | N/A | N/A | -0.06 (0.08) | 0.415 | -0.38 (0.11) | <0.001 |
| Per month: quadratic component | -0.25 (0.02) | <0.001 | -0.26 (0.02) | <0.001 | -0.28 (0.02) | <0.001 |
| Per frailty score: quadratic component | N/A | N/A | 0.01 (0.01) | 0.521 | 0.04 (0.02) | 0.005 |
|  |  |  |  |  |  |  |
|  | Variance (SD) |  | Variance (SD) |  | Variance (SD) |  |
| **Random effects** |  |  |  |  |  |  |
| At admission (intercept) |  |  |  |  |  |  |
| Between persons variance | 7.91 (2.81) |  | 5.00 (2.24) |  | 1.65 (1.29) |  |
| Between countries variance | 6.31 (2.51) |  | 2.16 (1.47) |  | 2.43 (1.56) |  |
|  |  |  |  |  |  |  |
| After admission (slope of change) |  |  |  |  |  |  |
| Between persons variance | 0.06 (0.25) |  | 0.04 (0.19) |  | 0.04 (0.19) |  |
| Between countries variance | 0.07 (0.26) |  | 0.07 (0.26) |  | 0.04 (0.19) |  |
|  |  |  |  |  |  |  |
| Residual | 10.45 (3.23) |  | 10.51 (3.24) |  | 10.00 (3.16) |  |
| *adjusted for: age, sex, premorbid BI, Functional Comorbidity Index, hospital length of stay, and ICU stay. | | | | | | |
